# BCL-2, topoisomerase IIα, microvessel density and prognosis of early advanced breast cancer patients after adjuvant anthracycline-based chemotherapy

**DOI:** 10.1007/s00432-014-1770-8

**Published:** 2014-07-09

**Authors:** Beata Biesaga, Joanna Niemiec, Marek Ziobro

**Affiliations:** 1 Department of Applied Radiobiology, Centre of Oncology, ul. Garncarska 11, 31-115 Kraków, Poland; 2 Department of Medical Oncology, Centre of Oncology, ul. Garncarska 11, 31-115 Kraków, Poland

**Keywords:** Early breast cancer patients, Anthracyclines, Potential prognostic factors

## Abstract

**Purpose:**

The aim of this retrospective study was to investigate the effect of B cell lymphoma 2 (BCL-2) expression on disease-free survival (DFS) in 172 early breast cancer (BC) patients treated with anthracycline-based adjuvant chemotherapy. We have reanalysed follow-up data in these patient groups, and therefore, the relation between DFS and other tumour biological features [expression of oestrogen (ER) and progesterone (PgR) receptors, cytokeratin 5/6 (CK5/6), HER2, topoisomerase IIα (TOPOIIα), Ki-67, P53 and microvessel density (MVD)] studied previously (Biesaga et al. in Breast 20(4):338–350, 2011, doi:10.1016/j.breast.2011.03.002, Pathol Oncol Res 18(4): 949–960, 2012, doi:10.1007/s12253-012-9525-9) was also investigated.

**Method:**

Tumour biological features were assessed immunohistochemically on paraffin-embedded sections obtained before treatment from 172 women with BC in stage T1–T2, N1–N2, M0.

**Results:**

In univariate analysis, longer DFS was found for patients having tumours with BCL-2 positivity (*P* = 0.005), low grade (*P* = 0.001), ER (*P* = 0.017) and PgR (*P* = 0.045) positivity, CK5/6 negativity (*P* = 0.021), low TOPOIIα expression (*P* = 0.003) and high MVD (*P* = 0.000). In multivariate analysis, BCL-2, TOPOIIα and MVD were independent parameters indicating patient prognosis. All patients (*n* = 18) characterized by tumour BCL-2 positivity, low TOPOIIα expression and high MVD survived 80 months without any evidence of cancer disease, whereas DFS for all other patients was significantly (*P* = 0.022) lower (76.5 %).

**Conclusion:**

Combination of three parameters: BCL-2 positivity, low topoisomerase IIα expression and high MVD, allows to identify subgroup of BC patients with very good prognosis after adjuvant anthracycline-based chemotherapy.

## Introduction

Adjuvant systemic therapy of patients with early advanced breast cancer (BC) significantly reduces recurrence risk and overall mortality. Anthracyclines (ATC) are the most commonly used drugs in the adjuvant chemotherapy (CT) of breast cancer. The last meta-analysis of Early Breast Cancer Trialists’ Collaborative Group demonstrated that regimens based on higher dosages of ATC are associated with around 4 % reduction of recurrence rate and overall mortality as compared with cyclophosphamide, methotrexate and fluorouracil scheme (CMF) (EBCTCG et al. 2012). There is a possibility to improve treatment outcome by more differentiated therapy, tailored to the biological characteristics of the tumour cells. For this purpose, it is necessary to undertake translational researches, consisting in the development of biological prognostic and predictive factors for adjuvant chemotherapy based on ATC. Today, there is no reliable prognostic factor which allows discrimination between patients who will benefit from adjuvant ATC treatment and those who will not. In our previous papers, in the group of 167 patients with breast cancers in clinical stage T1–T2, N1–N2, M0, treated with ATC in adjuvant setting, we have shown prognostic potential of topoisomerase IIα (TOPOIIα) expression (Biesaga et al. 2011) and tumour microvessel density (MVD) (Biesaga et al. 2012). However, chemotherapeutic agents such as ATC, irrespective of their intracellular target, act through induction of apoptosis in cancer cells. Therefore, alterations in the regulatory mechanism of apoptosis are responsible not only for cancer progression, but also for different response to treatment.

Antiapoptotic protein B cell lymphoma 2 (BCL-2) is one of the most extensively studied apoptosis regulators in translational studies, but its role in ATC resistance/sensitivity is unclear. There are a few reports (see Table 1), in which prognostic potential of BCL-2 expression was investigated in the breast cancer patient groups consisting of subgroups of women treated with ATC in adjuvant setting, although in all these studies, expect one (Abdel-Fatah et al. 2010) not analysed separately. In these studies, contradictory results were obtained. Moreover, in two meta-analyses, performed to investigate BCL-2 prognostic potential, opposite results were reported. Callagy et al. (2006) have shown positive prognostic significance of BCL-2 overexpression, whereas Yang et al. (2013) have found significant relation between BCL-2 negativity and good prognosis. These contradictions may be the effect of at least four causes: (1) other than antiapoptotic function of BCL-2 [the influence on cell cycle progression (Zinkel et al. 2006; Pietenpol et al. 1994)], (2) regulation of BCL-2 expression through oestrogen pathway (Perillo et al. 2000), (3) lack of homogeneity in patient group studied (according to clinical stage and adjuvant treatment type) and (4) differences in IHC procedure and cut-off points used to identified BCL-2 positivity and negativity (Table 1). Thus, there is still a need to evaluate the prognostic role of BCL-2 in BC patients.

**Table 1:** Studies on BCL-2 prognostic potential for adjuvant anthracycline-based chemotherapy

References	*N*	Adjuvant chemotherapy type	Cut-off point for BCL-2 immuno-positivity/overexpression	BCL-2 prognostic significance
van Slooten et al. (1996)	423	202 pts: no adjuvant CT 221 pts: FAC	>75 % of cells with moderate or strong intensity	BCL-2 positivity: better DFS, lack of significance in multivariate analysis
Mottolese et al. (2000)	157	4 cycles of EC or 4 cycles of EC + lonidamine or 4 cycles of EC + G-CSF or 4 cycles of EC + lonidamine + G-CSF	>20 % of stained cells	In the entire cohort: no significance in lobular carcinomas: BCL-2 positivity: worse OS and DFS
Yang et al. (2003)	147	All pts: FEC		BCL-2 positivity: better OS and DFS confirmed in multivariate analysis
Kröger et al. (2006)	157	78 pts: 3 cycles of CMF 79 pts: 4 cycles of EC → CTM → stem cell support	>0 % of stained cells	BCL-2 positivity: better EFS, confirmed in multivariate analysis
Lee et al. (2007)	151	AC → T	>0 % of stained cells	BCL-2 positivity: better OS and DFS confirmed in multivariate analysis
Dumontet et al. (2010)	1,342	663 pts: FAC 679 pts: TAC	>70 % of stained cells	BCL-2 positivity: better OS and DFS, lack of significance in multivariate analysis
Abdel-Fatah et al. (2010)	1,235	377 pts: no adjuvant treatment 382 pts: hormonal therapy 182 pts: CMF 32 pts: CMF + hormonal therapy 245 pts: anthracyclines	>10 % of stained cells	The entire patient cohort and separately patients treated with anthracyclines: BCL-2 positivity: better DFS and BCSS confirmed in multivariate analysis
Kim et al. (2010)	100	24 pts: no adjuvant treatment 30 pts: CMF 27 pts: doxorubicin containing CT 3 pts: others chemotherapy 16 pts: hormonal therapy only	>10 % of stained cells	No significance

*pts* patients, *CT* chemotherapy, *FAC* 5-fluorouracil and doxorubicin and cyclophosphamide, *DFS* disease-free survival, *EC* epirubicin and cyclophosphamide, *OS* overall survival, *CMF* cyclophosphamide and methotrexate and 5-fluorouracil, *CTM* cyclophosphamide and tiotepa and mitoxantrone, *EFS* event free survival, *AC* doxorubicin and cyclophosphamide, *T* paclitaxel, *TAC* docetaxel and doxorubicin and cyclophosphamide, *BCSS* breast cancer-specific survival

The aim of the present study was to evaluate the prognostic significance of BCL-2 expression in the group of 172 women with breast cancer in clinical stage T1–T2, N1–N2, M0, treated with ATC in adjuvant settings. For this purpose, we assessed the correlation between this parameter and patient disease-free survival (DFS). Because in the present patient group we reanalysed follow-up data, we decided to assess once again the influence of all clinical (patients’ age tumour size, lymph node metastases, grade) and biological features (expression of steroid hormone receptors, cytokeratin 5/6 (CK5/6), HER2, TOPOIIα, Ki-67, P53 and MVD), which were previously studied (Biesaga et al. 2011, 2012) on DFS of breast cancer patients.

## Materials and methods

### Patients

This study was performed in the group of 172 patients with breast cancer who met the following criteria: (1) invasive ductal breast cancer in clinical stage T1–T2, N1–N2, M0, (2) radical surgery (mastectomy or breast conserving therapy), (3) adjuvant anthracycline-based chemotherapy (according to two regimes: FAC—5-fluorouracil, doxorubicin, cyclophosphamide or AC—doxorubicin, cyclophosphamide). All other details concerning this patient group were presented previously (Biesaga et al. 2011, 2012).

The study has been approved by the Ethics Committee at the Centre of Oncology, Krakow, Poland (date of issue 14.02.2006).

### Tumour samples

Formalin-fixed, paraffin-embedded tissue blocks were obtained from each patient, and serial 4-µm sections were processed for application of the immunohistochemistry (IHC).

### Immunohistochemistry

In order to assess BCL-2 expression, IHC staining was performed on 5-μm-thick sections from routinely fixed paraffin-embedded blocks. To unmask antigen, the deparaffinized (trough xylene series) and rehydrated sections (in decreasing concentrations of ethanol) were incubated for 50 min. in TRS (pH 6.1, DAKOCytomation, Glostrup, Denmark) preheated to 96 °C. Endogenous peroxidases were quenched by 30 min. incubation in 3 % hydrogen peroxide in 70 % methanol. Whole night incubation with diluted primary (1:40) antibody (Monoclonal Mouse Anti-Human, BCL-2 Oncoprotein, Clone 124, DAKOCytomation, Glostrup, Denmark) at 4 °C in humidity chamber was carried out (Table 2). The antigen-primary antibody immunoreaction was detected by BrightVision Poly- HRP-Anti Ms/Rb/Rt IgG (ImmunoLogic, Duiven, the Netherlands). For this purpose, after three times washing in TBS-T, the slides were incubated at room temperature (RT) with post-antibody blocking for 15 min and with poly-HRP-goat anti-mouse/rabbit IgG for 30 min. Peroxidase was visualized using 0.01 % 3.3-diaminobenzidine tetrahydrochloride (DAB) and 0.015 % hydrogen peroxide. The slides were counterstained with Mayer’s haematoxylin. Tumour specimen with known strong BCL-2 expression added to each series of staining was treated as positive control. For negative control, TBS was substituted for the primary antibody.

**Table 2:** Details of immunohistochemistry staining

Antigen	Clone	Source	Dilution	Detection system	Criteria for IHC positivity/overexpression
ER	6F11	Novocastra^a^	1:50	EnVision^b^	>10 % of tumour immunopositive cells
PgR	1A6	Novocastra^a^	1:50	EnVision^b^	>10 % of tumour immunopositive cells
HER2	–	DAKO^b^	1:250	EnVision^b^	Strong complete uniform membrane staining in >30 % of tumour cells or complete membrane staining in >10 % of tumour cells confirmed by FISH
CK 5/6	D5/16 B4	DAKO^b^	1:50	EnVision^b^	>10 % of tumour immunopositive cells
TOPOIIα	3F6	Novocastra^a^	1:30	EnVision^b^	TOPOIIα labelling index (TOPOIIαLI—the percentage of TOPOIIα immunopositive tumour cells) >11.9 % (median value)
Ki-67	MIB-1	DAKO^b^	1:75	EnVision^b^	Ki-67 labelling index (Ki-67LI—the percentage of Ki-67 immunopositive tumour cells) >19.7 % (median value)
CD34	QBEnd 10	DAKO^b^	1:50	EnVision^b^	High microvessel density (MVD—mean number of microvessels per mm^2^ of tumour tissue) >210.0 vessels/mm^2^ (cut-off point found by minimal *P* value method)
P53	NCL-P53-1801	Novocastra^a^	1:40	EnVision^b^	P53 labelling (index (P53LI—the percentage of P53 immunopositive tumour cells) >10.0 %
BCL-2	124	DAKO^b^	1:40	BrightVision^c^	Intense staining in all tumour cells (class 2) or heterogeneous staining within tumour area, regardless of the intensity (class 1)

*IHC* immunohistochemistry, *ER* oestrogen receptor, *PgR* progesterone receptor, *HER2* human epidermal growth factor receptor 2, *FISH* fluorescence in situ hybridization; *CK5/6* cytokeratin 5/6, *TOPOII*α topoisomerase IIα

^a^Laica Biosystems Newcastle Ltd, Newcastle, UK

^b^DakoCytomation Denmark A/S, Glostrup, Denmark

^c^ImmunoLogic, Duiven, the Netherlands

**Fig. 1:**
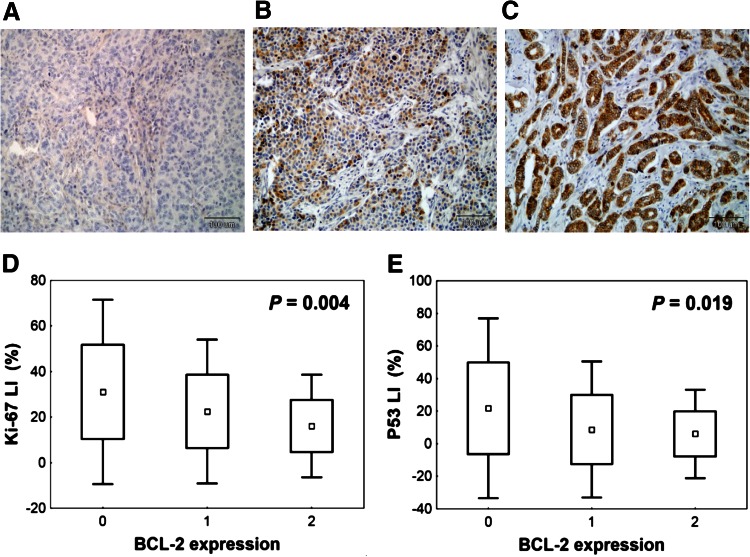
Representative images of BCL-2 immunohistochemical staining in breast cancer tissue and correlation between BCL-2 immunoexpression and other biological features studied. **a** Negative BCL-2 staining (class 0—no BCL-2 expression). **b** Heterogeneous BCL-2 immunostaining within tumour area (class 1—BCL-2 expression). **c** Intense BCL-2 staining in all tumour cells (class 2—BCL-2 overexpression), ×200 magnification. BCL-2 overexpression is correlated with **d** low proliferation rate assessed by Ki-67 labelling index (Ki-67LI), **e** low P53 level expressed as P53 labelling index (P53LI) (Kruskal–Wallis test)

Samples were analysed using Olympus microscope at 400× magnification. BCL-2 expression was scored according to classification presented by Treré et al. (2007), because based on it, they found significant relation between BCL-2 expression assessed by IHC and its mRNA level. According to this scale, three classes of BCL-2 expression were identified: 0—lack of immunostaining (no BCL-2 expression), 1—heterogeneous staining within tumour area, regardless of the intensity (BCL-2 expression) and 2—intense staining in all tumour cells (BCL-2 overexpression) (Fig. 1a–c).

All details regarding immunohistochemical analysis of steroid hormone receptors, CK5/6, HER2, TOPOIIα, Ki-67, P53 expression and MVD have been previously described (Biesaga et al. 2011, 2012) and in the present paper summarized in Table 2.

Breast cancer immunophenotypes were identified on the basis of ER, PgR, HER2 and Ki-67 labelling index (Ki-67LI) according to St. Gallen Expert Consensus (Goldhirsch et al. 2013 **):** luminal A-like (LA): ER and PgR positive, HER2 negative and Ki-67 “low” (in the present cohort ≤19.7 %), luminal B HER2^−^ (LBHER2^−^): ER positive, HER2 negative and at least one of: Ki-67 “high” (in the present cohort >19.7 %) or PgR negative, luminal B HER2^+^ (LBHER2^+^): ER positive, HER2 overexpressed or amplified, any Ki-67, any PgR, HER2^+^: HER2 overexpressed or amplified, ER and PgR absent, triple negative (TN): ER and PgR absent, HER2 negative.

### Statistical methods

All statistical analyses were carried out using Statistica v.10.0 program (StatSoft, USA). Descriptive statistics were used to determine mean and median values of continuous variables and standard errors of means (SE). The significance of differences in continuous variables between groups was assessed on the basis of Mann–Witney *U* test. Independence of categorical variables expressed in a cross-tab was analysed by Pearson’s chi-square test. Cumulative DFS probabilities were estimated using the Kaplan–Meier method, and the significance of differences between survival rates was calculated by the log-rank test. Multivariate analysis was carried out using the Cox proportional hazards model, providing an estimate of the hazard ratio (HR) and a 95 % confidence interval (95 % CI). *P* value <0.05 was considered to be significant.

## Results

### Patients

A 172 patients with invasive ductal breast cancer were analysed (Table 3). Women aged from 32 to 78 years (with mean and median values 52.8 years ± 0.67 and 53 years, respectively). Among these, 54.7 % were in clinical stage T1N1, 23.3 % in stage T1N2, 18.0 % in T2N1 and 4.0 % in T2N2.

**Table 3:** Clinical and biological features of breast cancer patients stratifying according to BCL-2 expression

	BCL-2 expression				
	All (%)^1^	0	1	2	*P* value^a^
		*N* (%)*	*N* (%)*	*N* (%)*	
All (%)	141 (100)	38 (27.0)	70 (49.6)	33 (23.4)	
Age					
<50 years	47 (33.3)	8 (17.0)	29 (61.7)	10 (21.3)	0.092
≥50 years	94 (66.7)	30 (31.9)	41 (43.6)	23 (24.5)	
Tumour size					
T1	38 (27.0)	9 (23.7)	17 (44.7)	12 (31.6)	0.378
T2	103 (73.0)	29 (28.1)	53 (51.5)	21 (20.4)	
Nodal status					
N1	108 (76.6)	26 (24.1)	56 (51.8)	26 (24.1)	0.376
N2	33 (23.4)	12 (36.4)	14 (42.4)	7 (21.2)	
Grade					
G1 + G2	71 (50.4)	10 (14.1)	37 (52.1)	24 (33.8)	0.000
G3	70 (49.6)	28 (40.0)	33 (47.1)	9 (12.9)	
Oestrogen receptor status					
Positive	97 (68.8)	11 (11.0)	58 (58.0)	31 (31.0)	0.000
Negative	44 (31.2)	28 (63.6)	14 (31.8)	2 (4.6)	
Progesterone receptor status					
Positive	93 (66.0)	10 (10.7)	53 (57.0)	30 (32.3)	0.000
Negative	48 (34.0)	28 (58.3)	17 (35.4)	3 (6.3)	
Cytokeratin 5/6 expression					
Positive	30 (21.3)	13 (43.3)	15 (50.0)	2 (6.7)	0.015
Negative	111 (78.7)	25 (22.5)	55 (49.6)	31 (27.9)	
HER2 status					
Overexpressing	54 (38.3)	19 (35.2)	27 (50.0)	8 (14.8)	0.084
Not overexpressing	87 (61.7)	19 (21.8)	43 (49.4)	25 (28.7)	
TOPOIIαLI^c^					
≤11.9 %	71 (50.4)	16 (22.5)	37 (52.1)	18 (25.4)	0.486
>11.9 %	70 (49.6)	22 (31.4)	33 (47.1)	15 (21.4)	
Ki-67LI^c^					
≤19.7 %	74 (52.5)	15 (20.3)	36 (48.6)	23 (31.1)	0.038
>19.7 %	67 (47.5)	23 (34.3)	34 (50.7)	10 (14.9)	
MVD^d^					
≤210.0 vessels/mm^2^	96 (68.1)	28 (29.1)	47 (49.0)	21 (21.9)	0.645
>210.0 vessels/mm^2^	45 (31.9)	10 (22.2)	23 (51.1)	12 (26.7)	
P53LI					
≤10.0 %	100 (70.9)	20 (20.0)	55 (55.0)	25 (25.0)	0.014
>10.0 %	41 (29.1)	18 (43.9)	15 (36.6)	8 (19.5)	
Breast cancer immunophenotypes^e^					
LA	42 (29.8)	5 (11.9)	19 (45.2)	18 (42.9)	0.000
LB HER2^−^	22 (15.6)	0 (0.0)	16 (72.7)	6 (27.3)	
LB HER2^+^	33 (23.4)	5 (15.2)	21 (63.6)	7 (21.2)	
HER2^+^	21 (14.9)	14 (66.7)	6 (28.5)	1 (4.8)	
TN	23 (16.3)	14 (60.9)	8 (34.8)	1 (4.3)	

*HER2* human epidermal growth factor receptor 2, *TOPOII*α*LI* topoisomerase IIα labelling index, *Ki*-*67LI* Ki-67 labelling index, *MVD* microvessel density, *P53LI* P53 labelling index, *LA* luminal A, *LB* luminal B, *TN* triple negative

^1 ^Column percentage

* Row percentage

^a^ *P* values are from Pearson’s *χ* ^2^ test

^b^Mean value

^c^Median values

^d^Cut-off point from minimal *P* value method

^e^Immunophenotypes indicated on the basis of ER, PgR, HER2 and Ki-67 expression according to St. Gallen International Expert Consensus on The Primary Therapy of Early Breast Cancer 2013 (Goldhirsch et al. 2013)

Most of the patients (89.0 %) were subjected to mastectomy and 11 % underwent breast conserving surgery. All women received adjuvant anthracycline-based chemotherapy according to two regimes: six or four cycles of AC (59.9 % of patients) or six or four cycles of FAC (40.1 % of patients). The patients were assigned to these two regimes because in the Centre of Oncology, Krakow Branch, the period of time between 2001 and 2005, when patients were recruited for the study, was transition time in the use of FAC instead of AC for adjuvant anthracycline-based chemotherapy. Due to positive lymph nodes, all patients underwent locoregional radiotherapy (45 Gy/2 Gy/day + boost or 50 Gy/2 Gy/day) as soon as possible after last chemotherapy course. All women with ER or PgR positivity (77.9 %) were treated with tamoxifen for 5 years. Women included in the study did not receive trastuzumab, because in Poland, trastuzumab has been used and reimbursed by the National Health Fund for the adjuvant treatment since 2007.

All data regarding treatment outcome in this patient group were reanalysed in June 2012. In contrast to our earlier analyses (Biesaga et al. 2011, 2012) in which we obtained data for 167 patients, at present follow-up was available for 171 patients, one patient is still lost from follow-up. The mean and median length of follow-up were 61.8 months ± 1.9 (SE) and 69 months, respectively, (range 1–126 months). Tumour progression (locoregional recurrence, distant recurrence or second malignancy) was observed in 35 patients (20.5 %), after 1–70 months (mean and median values, respectively, 24.8 months ± 2.7 and 24.0 months) after surgery.

### BCL-2 expression, apoptosis level and correlations with clinicopathological features and other immunohistochemical markers

Due to accessibility of material in paraffin blocks in this series, the evaluation of BCL-2 expression was possible for 141 (82.0 %) out of 172 patients. According to BCL-2 expression, 38/141 (27.0 %) were classified as 0 (no expression), 70/141 (49.6 %) as 1 (BCL-2 expression) and 33/141 (23.4 %) as 2 (BCL-2 overexpression) (Table 3). BCL-2 overexpression was significantly related to lower tumour grades (*P* = 0.001), positive hormonal receptors status (*P* = 0.000) and CK5/6 negativity (*P* = 0.015). The significantly higher percentage of tumours overexpressing BCL-2 was also found in LA and LB than in HER2^+^ and TN cancer immunophenotypes, identified on the basis of ER, PgR, HER2 and Ki-67 expression according to St. Gallen International Expert Consensus on The Primary Therapy of Early Breast Cancer 2013 (Goldhirsch et al. 2013).

BCL-2 expression status significantly correlates with Ki-67LI (*P* = 0.038) and P53 labelling index (P53LI) (*P* = 0.014), both analysed as categorical variables. Ki-67LI was categorized using median value (19.7 %). In case of P53LI, we decided, similar to other authors (Malamou-Mitsi et al. 2006; Mottolese et al. 2000), to accept the threshold for the P53 positivity at the level of 10 %. The percentage of BCL-2 overexpressed tumours was significantly higher in lower proliferating cancers with P53 negativity. Similar relations between BCL-2 expression and Ki-67LI or P53LI were also observed, if these two variables were analysed as continuous variables (Fig. 1d, e).

There was also a tendency suggesting associations between BCL-2 expression and patient age (*P* = 0.092) or HER2 status (*P* = 0.084); however, these relations did not reach statistical significance.

### Univariate survival analysis

In the present patient cohort, the DFS was 78.8 %. Univariate analysis showed the highest DFS (87.9 %) for women having tumours with BCL-2 overexpression (class 2), while the lowest (64.9 %) for those with BCL-2-negative cancers (class 0) (Table 4). Patients with BCL-2 expression in class 1 were characterized by intermediate DFS (84.3 %). The differences in DFS according to BCL-2 expression were significant (*P* = 0.005).

**Table 4:** Univariate Cox proportional hazard model for disease-free survival of 171 breast cancer patients treated with adjuvant anthracycline-based chemotherapy

	Response *N* (%)	HR	95 % CI	*P* value*
Age				
>50 years	90/115 (78.3)	1.080	0.534–2.189	0.830
≤50 years	45/56 (80.4)	1.000		
Tumour size				
T1	45/52 (86.5)	1.000	0.846–4.374	0.109
T2	90/119 (75.6)	1.924		
Nodal status				
N1	107/132 (81.1)	1.000	0.783–3.214	0.206
N2	28/39 (71.8)	1.587		
Grade				
G1 + G2	81/92 (88.0)	1.000	1.508–6.198	0.001
G3	54/79 (68.4)	3.057		
Oestrogen receptor status				
Positive	102/123 (82.9)	1.000	0.262–1.021	0.017
Negative	33/48 (68.8)	1.932		
Progesterone receptor status				
Positive	98/119 (82.4)	1.000	0.296–1.150	0.045
Negative	37/52 (71.2)	1.716		
HER2 status				
Overexpressing	55/68 (80.9)	1.000	0.523–1.984	0.750
Not overexpressing	80/103 (77.7)	1.019		
Cytokeratin 5/6				
Positive	23/35 (65.7)	2.301	1.154–4.588	0.021
Negative	112/136 (82.4)	1.000		
Ki-67LI^a^				
≤19.7 %	74/90 (82.2)	1.000	1.063–4.329	0.270
>19.7 %	61/81 (75.3)	2.196		
TOPOIIαLI^a^				
≤11.9 %	73/83 (88.0)	1.000	1.376–5.886	0.003
>11.9 %	62/88 (72.6)	2.846		
MVD^b^				
>210.0 vessels/mm^2^	52/53 (98.1)	1.000	0.009–0.460	0.000
≤210.0 vessels/mm^2^	83/118 (71.3)	15.724		
P53 LI				
>10.0 %	35/50 (70.0)	1.935	1.000–3.742	0.052
≤10 %	100/121 (82.6)	1.000		
BCL-2 expression				
Class 0	24/37 (64.9)	2.873	0.156–0.776	0.005
Class 1	59/70 (84.3)	1.302		
Class 2	29/33 (87.9)	1.000	0.246–2.401	
Breast cancer immunophenotypes^d^				
LA	41/50 (80.8)	1.000	0.350–2.164	0.081
LB HER2^−^	24/29 (79.6)	1.084		
LB HER2^+^	37/44 (84.0)	1.094	0.386–2.410	
HER2^+^	18/24 (74.5)	1.717	0.624–4.729	
TN	15/24 (62.5)	2.581	1.024–6.503	

*HR* hazard ratio, *CI* confidence interval, *HER2* human epidermal growth factor receptor 2, *Ki*-*67LI* Ki-67 labelling index, *TOPOIIαLI* topoisomerase IIα labelling index, *MVD* microvessel density, *P53LI* P53 labelling index, *LA* luminal A, *LB* luminal B, *TN* triple negative

*P values from log-rank test

^a^Median value

^b^Cut-off point from minimal *P* value method

^c^Mean value

^d^Immunophenotypes indicated on the basis of ER, PgR, HER2 and Ki-67 expression according to St. Gallen International Expert Consensus on The Primary Therapy of Early Breast Cancer 2013 (Goldhirsch et al. 2013)

The significant differences in DFS according to BCL-2 expression classes were more apparent in the subgroup of younger patients (*P* = 0.0140), with T1 (*P* = 0.032), N1 (*P* = 0.033) tumours, carcinomas characterized by negativity of CK5/6 (*P* = 0.043), HER2 (*P* = 0.019) and P53 (*P* = 0.037), low MVD (*P* = 0.013) and only for patients with cancers having TN immunophenotype. In TN subgroup, women with BCL-2 immunopositive (classes 1 + 2) tumours were characterized by 85.7 % of DFS, whereas those without BCL-2 expression by 38.5 % of DFS. This difference was statistically significant (*P* = 0.048).

Since we have updated follow-up data, in the present analysis, we also re-examined the relation between DFS and previously evaluated biological features of tumours studied (Biesaga et al. 2011, 2012). For this analysis, in case of three continuous variables: Ki-67LI, TOPOIIαLI and MVD, we checked the level of cut-off point by minimum *P* value method from log-rank test. For Ki-67LI and TOPOIIαLI, the cut-off point values remained at the same level as previously published (median values for both parameters) (Biesaga et al. 2011, 2012). For MVD, we changed the cut-off point from 214.8 to 210.0 microvessels/mm^2^, as at this cut-off point, the highest level of significance was observed. DFS was significantly associated with histological grade (*P* = 0.001), ER (*P* = 0.017) or PgR (*P* = 0.045) status, CK5/6 expression (*P* = 0.021), TOPOIIαLI (*P* = 0.003) and MVD (*P* = 0.000) (Table 4). In case of P53 expression and cancer immunophenotypes, this relation was at significance border.

### Multivariate analysis

 The multivariate analysis included the variables for which in univariate analysis, significant differences or differences at significance border in DFS were seen, i.e. grade, hormone receptors status, CK5/6 expression, Ki-67LI, TOPOIIαLI, MVD, P53LI, BCL-2 expression and breast cancer immunophenotypes. BCL-2 expression was categorized into two classes: BCL-2 negativity (class 0) and positivity (classes 1 + 2). In case of breast cancer immunophenotypes, we stratified patients into two subgroups: those with LA and LB (HER2^−^ and HER2^+^) carcinomas and those with HER2^+^ or TN tumours. Multivariate analysis revealed topoisomerase IIα expression, microvessel density and BCL-2 status as independent prognostic factors for adjuvant chemotherapy with anthracyclines (Table 5).

**Table 5:** Multivariate Cox regression analysis on disease-free survival of 171 breast cancer patients

	HR	95 % CI	*P* value
Grade			
1 + 2	1.000	0.611–4.176	0.329
3	1.610		
Oestrogen receptor status			
Positive	1.000	0.580–3.792	0.412
Negative	1.484		
Progesterone receptor status			
Positive	1.000	0.355–1.748	0.560
Negative	1.269		
Cytokeratin 5/6 expression			
Positive	1.801	0.782–4.144	0.169
Negative	1.000		
TOPOIIαLI^a^			
≤11.9 %	1.000	1.202–6.303	0.017
>11.9 %	2.752		
MVD^b^			
>210.0 vessels/mm^2^	1.000	0.010–0.509	0.009
≤210.0 vessels/mm^2^	14.352		
P53LI			
≤10.0 %	1.000	0.536–2.679	0.662
>10.0 %	1.198		
BCL-2 expression			
Classes 1 + 2	1.000	0.153–0.699	0.004
Class 0	3.056		
Breast cancer immunophenotypes^d^			
LA + LB (HER2^−^ and HER2^+^)	1.000	0.322–2.886	0.947
HER2^+^ + TN	1.038		

*TOPOIIαLI* topoisomerase IIα labelling index, *MVD* microvessel density, *P53LI* P53 labelling index, *LA* luminal A, *LB* luminal B, *TN* triple negative

^a^Median values

^b^Cut-off point from minimal *P* value method

^c^Mean value

^d^Immunophenotypes indicated on the basis of ER, PgR, HER2 and Ki-67 expression according to St. Gallen International Expert Consensus on The Primary Therapy of Early Breast Cancer 2013 (Goldhirsch et al. 2013)

**Fig. 2:**
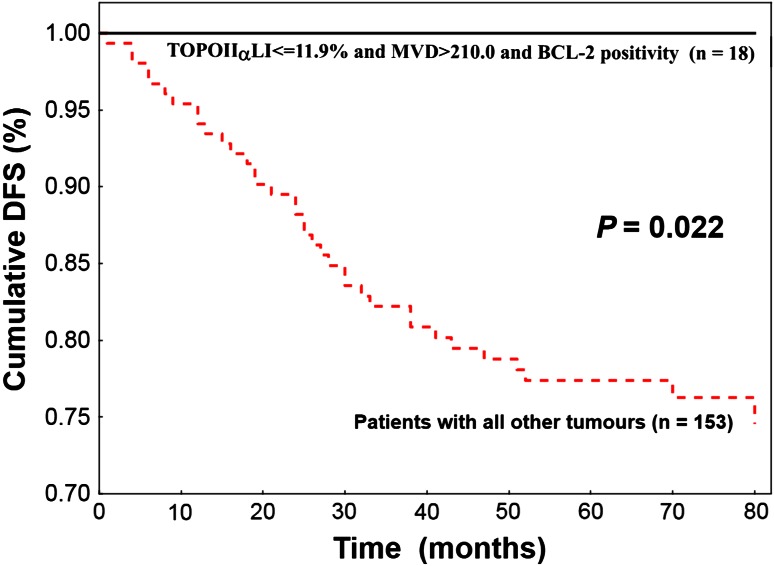
Low topoisomerase IIα expression, high microvessel density and BCL-2 positivity indicate 100 % probability of 5-year disease-free survival (DFS) of 18 patients with breast cancer. Women having tumours with other combination of these three biological features (*n* = 153) demonstrate significantly lower DFS (log-rank test)

Based on these results, we decided to divide the analysed group into two subgroups of different prognosis: (1) women with tumours characterized by low TOPOαLI and high MVD and BCL-2 positivity (*n* = 18) and (2) the remaining patients (*n* = 153). All 18 patients from subgroup 1 survived 80 months after treatment completion without any evidence of cancer disease, whereas DFS for women from subgroup 2 was 76.5 % (Fig. 2). The difference in DFS between these subgroups was significant (*P* = 0.022).

## Discussion

In the present study, we have shown, to the best of our knowledge, for the first time, 100 % DFS for patients having tumours characterized by low topoisomerase IIα expression and high microvessel density and BCL-2 positivity (all assessed by immunohistochemistry). The results suggest that these three parameters may constitute an input into the new prognostication model specially dedicated to adjuvant anthracycline-based chemotherapy of breast cancer patients. This suggestion is in agreement with other results showing that BCL-2 expression can improve prognostication of BC patients treated with different CT regimens. Abdel-Fatah et al. (2010), in the group of 245 patients with invasive BC treated with adjuvant ATC-based chemotherapy, found a good prognosis for patients with active or partially inactive P53 pathway (assessed by expression of BCL-2, p21, p27, hormone receptors, P53, Ki-67, EGFR, CK 5/6 and HER2 gene amplification status). In turn, Hwang et al. (2012) assessed prognostic influence of BCL-2 expression in the group of 7,230 primary BC patients in clinical stage 1, 2 or 3, of whom 4,532 received different systemic therapy. They have shown that the risk groups of cancer patients, identified by St. Gallen model or Nottingham Prognostic Index model or TNM staging, could be subdivided by addition of the BCL-2 assessment in all three models. Callagy et al. (2006) have also found that BCL-2 was the only marker from a panel of 13 proteins (HER2, c-Myc, cyclin E, CK5/6, CK17, CK8/18, ER, Ki-67, MCM-2, p27, P53 and PgR) that improved prognostication in the group of 930 breast cancer patients, some of whom were treated with adjuvant CT.

The positive prognostic significance of low TOPOIIα expression and high MVD is in concordance with results obtained by some authors (TOPOIIα: Fritz et al. 2005; Depowski et al. 2000; MVD: Gunel et al. 2002; Protopapa et al. 1993). The positive effect of low TOPOIIα may be explained by hypothesis suggesting that low enzyme expression is related to less aggressive tumour phenotype, characterized by low histological grade, hormone receptors positivity, CK5/6 negativity and low proliferation rate, what was shown by us (Biesaga et al. 2011) and other authors (Fritz et al. 2005; Durbecq et al. 2004; Depowski et al. 2000). In turn, the positive influence of high MVD on ATC treatment outcome may be related to enhancement of production of reactive oxygen species under ATC or increased drug access to tumour cells in well-vascularized tumours. All these suggestions were carefully discussed in our earlier publications (Biesaga et al. 2011, 2012); therefore, in the present paper, we would like to focus on prognostic potential of BCL-2 expression.

We have shown that BCL-2 expression is independent prognostic factor for adjuvant chemotherapy with ATC, what is in agreement with results of a few other reports in which analysis of subgroups of patients treated with ATC in adjuvant settings was provided (see Table 1). Some of these data were summarized in meta-analysis performed by Callagy et al. (2008), who included seventeen papers published since 2006, reporting data of almost 6,000 patients with BC, regardless of the treatment type. This meta-analysis supports that BCL-2 overexpression indicates good prognosis. This effect was independent of lymph node status, tumour size or grade as well as a range of other biological variables. However, another meta-analysis from 2013, in which 23 studies involving almost 2,500 BC patients, showed that negative BCL-2 expression was associated with pathological complete response in women treated with ATC-based neoadjuvant chemotherapy (Yang et al. 2013). We mentioned about the possible causes of these contrary results in Introduction section.

The reasons of positive prognostic potential of BCL-2 overexpression are not fully understood. It is well known that this protein has oncogenic function related to inhibition of apoptosis and hence tumour progression and metastatic potential (Short and Johnstone 2012). However, in some types of tumour, BCL-2 seems to play both oncogenic and suppressive role. It is postulated that the dominance of one of these functions over another may depend on the cell type and physiology and that suppressive effect dominates in solid epithelial tumours including breast cancer (Zinkel et al. 2006). BCL-2 suppressive function may be a consequence of its non-apoptotic functions, particularly its ability to control cell cycle machinery, what was supported by cell line studies in which relation between BCL-2 expression and G0 prolongation and delay of G1-S transition was shown (Zinkel et al. 2006; Pietenpol et al. 1994).

Positive prognostic significance of BCL-2 overexpression may be explained by its influence on BC cell chemosensitivity. Del Bufalo et al. (2002) have shown that human BC cell lines overexpressing BCL-2 displayed an increased sensitivity to drugs known to induce multidrug resistance phenomenon (MDR) such as doxorubicin, vincristine, vinblastine and actinomycin, whereas they show increased resistance to drugs not related to the MDR (cisplatin and bischloroethylnitrosourea). The enhanced sensitivity to doxorubicin was associated with an increased drug accumulation and its decreased efflux as well as with reduction of the adenosine triphosphate level and kinase C activity, both of which participate in the regulation of MDR. In earlier studies, the above-referred authors have demonstrated that BCL-2 overexpression was related to inhibition of mitochondrial metabolism and reduction of oxygen uptake and ^14^CO_2_ production (Biroccio et al. 1999).

The positive prognostic power of BCL-2 overexpression may be also related to its correlation with other favourable prognostic markers such as lower tumour grade, oestrogen and progesterone positivity, cytokeratin negativity, lack of HER2 overexpression, which was shown in our study and is in agreement with other authors (van Slooten et al. 1996; Mottolese et al. 2000; Yang et al. 2003; Lee et al. 2007; Kim et al. 2010). We have also found the higher percentage of BCL-2-positive tumours among luminal A and B cancer immunophenotypes as compared with HER2^+^ and TN immunophenotypes. Therefore, BCL-2 expression may also reflect lower degree of tumour differentiation.

In the presented analysis, based on BCL-2 expression, we could stratify patients with triple negative tumours into two subgroups with different prognosis. TN breast cancer patients with BCL-2 overexpression had significantly higher DFS than those with BCL-2 negativity. These results should be, however, interpreted with care, because of the small number of patients in each subgroup (7 and 13, respectively). Nonetheless, presented results are partly in agreement with those obtained by other authors. Abdel-Fatah et al. (2013) in the large cohort of 635 patients with early advanced TN breast cancers have found that BCL-2 negativity was associated with approximately twice the risk of death and recurrence in patients who did not receive CT or were treated with CMF. In turn, they have shown longer survivals in BCL-2-negative TN BC patients exposed to anthracyclines than in those who did not receive chemotherapy or were treated with CMF. Relatively high DFS in BCL-2-positive TN tumours obtained by us could also confirm results of earlier reports, showing that a subset of TN breast cancer patients responded to standard chemotherapy and had survival rate similar to women with other breast cancer immunophenotypes (Liedtke et al. 2008). However, Tawfik et al. (2012), contrary to us, have demonstrated association between BCL-2 positivity and poorer survival only among non-TN breast cancer patients, whereas in the group of TN breast cancer patients, BCL-2 status had no influence on treatment outcome. These discrepancies may be explained by heterogeneity of TN subtype, which represents distinct molecular subgroups that differentially respond to chemotherapy. Lehmann et al. (2011), analysing gene expression profiles, have identified six distinct molecular subtypes of TNBC including two basal like, an immunomodulatory, a mesenchymal, a mesenchymal stem-like and a luminal androgen receptor subtype. In the next report of this team, in the group of 130 patients with TN BC who received neoadjuvant chemotherapy containing sequential taxane and ATC chemotherapy, significant relation between TN BC subtypes and pathological complete response status was found (Masuda et al. 2013). Thus, in the light of our and some other author’s results, we postulate that BCL-2 expression may be one of the surrogate markers allowing distinguishing TN BC subtypes with different sensitivity to ATC; however, this supposition requires confirmation in further studies.

In conclusion, in the present study, we have shown, to the best of our knowledge for the first time, that by combination of three parameters: BCL-2 positivity, low topoisomerase IIα expression and high microvessel density, it is possible to identify subgroups of BC patients with very good prognosis after adjuvant anthracycline-based chemotherapy. All these three parameters were assessed by immunohistochemistry, which is an inexpensive and routine method, widely used in pathology and radiobiology departments. Therefore, assessment of these proteins is easy for implementation in daily clinical practise. However, the presented results should be confirmed in larger, prospective clinical trials.
